# Novel amperometric glucose biosensor based on MXene nanocomposite

**DOI:** 10.1038/srep36422

**Published:** 2016-11-10

**Authors:** R. B. Rakhi, Pranati Nayuk, Chuan Xia, Husam N. Alshareef

**Affiliations:** 1 Materials Science and Engineering, King Abdullah University of Science and Technology, (KAUST), Thuwal 23955-6900, Saudi Arabia; 2 Chemical Sciences and Technology Division, CSIR-National Institute of Interdisciplinary Science and Technology (CSIR-NIIST), Thiruvananthapuram, 695019, India

## Abstract

A biosensor platform based on Au/MXene nanocomposite for sensitive enzymatic glucose detection is reported. The biosensor leverages the unique electrocatalytic properties and synergistic effects between Au nanoparticles and MXene sheets. An amperometric glucose biosensor is fabricated by the immobilization of glucose oxidase (GOx) enzyme on Nafion solubilized Au/ MXene nanocomposite over glassy carbon electrode (GCE). The biomediated Au nanoparticles play a significant role in facilitating the electron exchange between the electroactive center of GOx and the electrode. The GOx/Au/MXene/Nafion/GCE biosensor electrode displayed a linear amperometric response in the glucose concentration range from 0.1 to 18 mM with a relatively high sensitivity of 4.2 μAmM^−1^ cm^−2^ and a detection limit of 5.9 μM (S/N = 3). Furthermore, the biosensor exhibited excellent stability, reproducibility and repeatability. Therefore, the Au/MXene nanocomposite reported in this work is a potential candidate as an electrochemical transducer in electrochemical biosensors.

MXenes, a new family of two-dimensional (2D) metal carbides, have attracted significant research interest in recent years due to their unique properties such as metallic conductivity and hydrophilic surfaces leading to stability in aqueous environments^1^,^2^. This rare combination of properties makes them suitable candidates for various promising applications. MXenes are synthesized by the selective etching of the “A” layers from the layered carbides known as MAX phases, resulting in a morphology similar to the exfoliated graphite^3^. These materials can be reliably produced in large quantities, which guarantees their scalable production for various applications^4^. The potential use of MXenes as efficient electrode materials for electrochemical energy storage applications has been well demonstrated^5^,^6^,^7^,^8^. MXenes present great application prospects as electrochemical transducer in sensing applications as well^9^.

The detection and quantification of glucose- the most important monosaccharide in vital life processes- are popular research topics in the field of sensors^10^,^11^,^12^,^13^,^14^,^15^. Electrochemical sensing, by virtue of its rapid response, ease of use, lowcost, portability, reliability, good sensitivity, and excellent selectivity, is one of the promising and widely accepted technique for glucose sensing^12^,^15^,^16^,^17^. Recognition element, electrochemical transducer and a signal processing unit for the recording, amplification and user-friendly data representation are the three major components of an electrochemical biosensor^18^,^19^. Recognition element, the primary component of a biosensor, allows the sensor to respond selectively to one particular analyte from among a large number of other substances. Depending on the type of recognition element, biosensors can be classified as enzymatic, non-enzymatic, whole-cell and immunosensors^20^. Among these, enzymatic biosensors are highly selective, sensitive, fast and reversible^21^,^22^. Glucose biosensor is the most common enzymatic biosensor. The enzyme used for the detection of glucose is glucose oxidase (GOx)^23^,^24^,^25^,^26^,^27^. GOx catalyses the oxidation of glucose into gluconolactone and hydrogen peroxide^28^.

An efficient and sensitive glucose biosensor with good sensitivity can be fabricated by immobilizing GOx with an appropriate electrochemical transducer^21^,^24^,^29^,^30^,^31^,^32^. Physical adsorption (drop casting method) is the simplest of all the immobilizing techniques to incorporate enzymes to transducers without affecting their native conformation, and this technique is commonly used for biosensors in the research stage^33^. Being a mechanically stable, compact, solid, impermeable to gasses and liquids, glassy carbon electrode (GCE) is a widely accepted biosensor immobilization matrix^34^,^35^,^36^. However, the potential for the oxidation/reduction of H_2_O_2_ over GCE is relatively high. This potential can be lowered by the incorporation of biocompatible and highly conductive Au nanoparticles onto the electrochemical transducer immobilization matrix^36^,^37^,^38^. When used as a component in enzymatic electrochemical biosensors, Au nanostructures allow enzymes (proteins) to retain their biological activity upon adsorption and reduce the insulating effect of the protein shell for direct electron transfer^21^,^23^,^39^,^40^,^41^,^42^,^43^,^44^,^45^. The performance of an electrochemical biosensor (sensitivity, selectivity, detection limit, detection potential, linearity range) essentially depends on the specific surface area, conductivity, charge transfer properties and more importantly the catalytic activity of the sensing matrix. Materials having high specific surface area build up more current, which improve significantly the sensitivity and linearity of a biosensor^46^. Similarly materials having better catalytic activity lower the detection potential, improve sensitivity and detection limit^47^. In this respect, nanomaterials having different size, shape and crystal facets influence the performance of the sensor. With decreasing size of a metal nanoparticle, the effect of atoms at corners and edges of nanoparticles becomes dominant which exhibit intrinsically different catalytic performance in contrast to a nanoparticle with a larger size^48^. Similarly, materials with different shapes (flowers, sheets) usually exhibit quite different catalytic behaviors intrinsically resulting from the differences in geometric structure and electronic state of the catalyst atoms associated with different crystallographic surfaces^49^. Ishida *et al*. studied the effect of size of gold nanocluster on catalytic performance in the oxidation of glucose^50^. Many reports are available on the efficient use of Au/nanocarbon composite as electrochemical transducer in glucose biosensors^37^,^38^. Reports also suggest the extensive use of Nafion solution for the better adhesion of enzyme molecules to the GCE^31^,^51^,^52^.

To the best of our knowledge, no reports are available on the use of Au/MXene composites for biosensors applications. In this paper, we report for the first time the fabrication and analytical performance of an enzymatic glucose biosensor electrode by the deposition of 32 units of GOx onto Nafion solubilized Au/MXene nanocomposite over GCE. Ti_3_C_2_T_x_ MXene nanosheets are prepared by the selective etching of Al from the Ti_3_AlC_2_ MAX phase. Crystalline Au nanoparticles are anchored onto the surface of the MXene sheets by a simple chemical reduction method. The performance of the biosensor is investigated by different electrochemical techniques, and the results are discussed.

## Results

### Characterization and morphology of the electrochemical transducers

**Figure 1:**
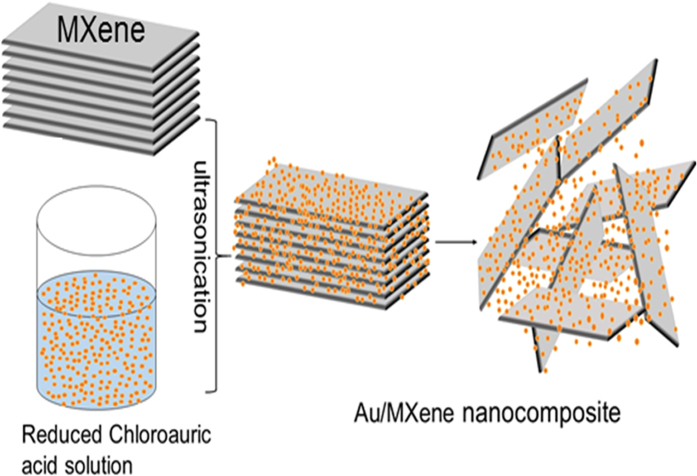
Schematic showing the synthesis process of Au/MXene nanocomposites.

**Figure 2:**
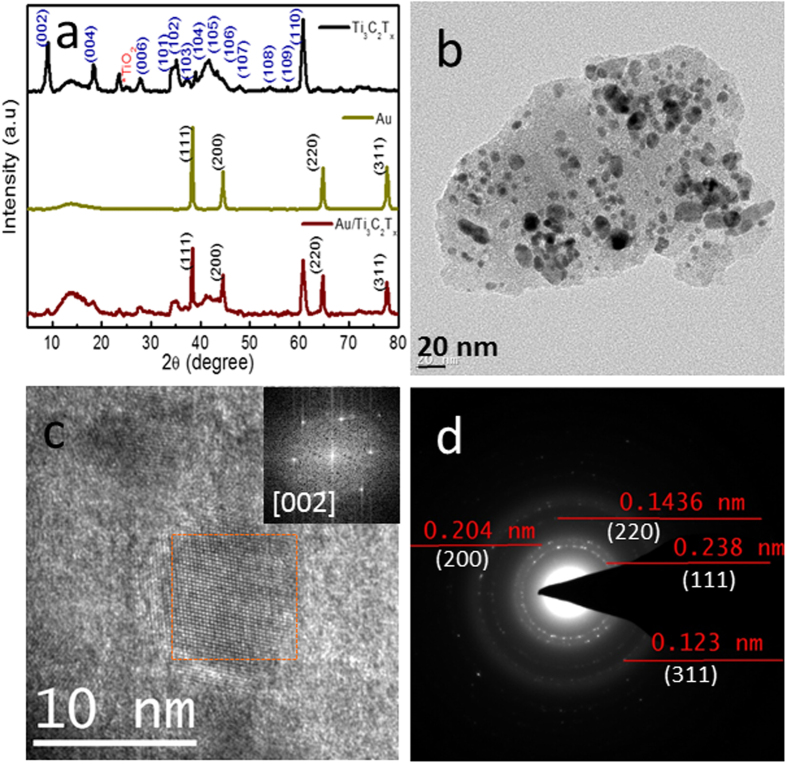
(**a**) Powder X-ray diffraction pattern of Ti_3_C_2_T_x_ MXene, Au nanoparticles and Au/MXene nanocomposite (**b**) TEM and (**c**) HRTEM images of Au/MXene nanocomposite. Inset of (**c**) shows the FFT pattern taken from the marked area. (**d**) SAED pattern corresponding to the marked area in (**c**).

The schematic of the synthesis of Au/MXene composite is shown in Fig. 1. Powder XRD patterns of Ti_3_C_2_T_x_ MXene, Au nanoparticles and Au/MXene nanocomposite are shown in Fig. 2a. The pattern of Ti_3_C_2_T_x_ MXene agrees well with XRD pattern of HF treated Ti_3_C_2_T_x_ in the literature. All the diffraction peaks in the XRD pattern of the nanoparticles collected from the solution can be readily indexed to a pure face-centered cubic phase of Au (space group: Fm3m (225), JCPDS card no. 00-004-0784). The XRD pattern of Au-MWNTnanocomposite material shows the reflections of Au along with that of Ti_3_C_2_T_x_ MXene. Extensive TEM analysis was carried out to investigate the microstructure of the Au/MXene nanocomposite in detail. TEM image of Au/MXene composite (Fig. 2b) shows an almost uniform distribution of Au nanoparticles over the surface of an exfoliated extremely thin and transparent MXene nanosheet. Ultrasonication results in the separation of the individual MXene sheet from the stack. The average particle size of Au is 6–8 nm. Majority of the Au nanoparticles remain attached to the MXene sheets, even after the TEM specimen preparation process involving ultrasonication, indicating that strong interactions exist between the Au nanoparticles and the MXene nanosheets. Fast Fourier transform (FFT) pattern of a selected region, from the HRTEM image of Au/MXene composite shown in Fig. 2c, obtained along the [002] zone axis is shown as inset of Fig. 2c and the corresponding selected area electron diffraction (SAED) pattern is shown in Fig. 2d. The SAED pattern indicates the polycrystalline structure of the nanogold. Diffraction rings in the SAED pattern can be indexed to the FCC cubic Au structure using circular Hough diffraction analysis, and these results agree well with the PXRD results. The HRTEM image in Fig. 2c clearly displays the crystalline nature of the Au nanoparticles.

### Electrochemical characterization of fabricated glucose biosensors

**Figure 3:**
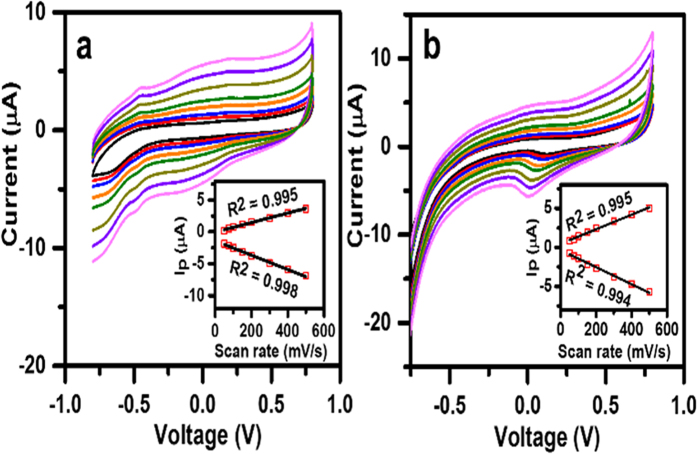
CV profile of (**a**) GOx/Au/MXene/Nafion/GCE and (**b**) GOx/MXene/Nafion/GCE bioelectrodes in N_2_-saturated PBS solution (0.1 M, pH7.0) at different scan rates: 50–500 mV/s. Insets: Plot of corresponding redox peak currents (Ip) vs. square root of scan rates.

For evaluation of the electron transfer phenomenon of the fabricated biosensor electrodes, cyclic voltammetric (CV) responses were recorded for the GOx/MXene/Nafion/GCE and GOx/Au/MXene/Nafion/GCE bioelectrodes at different scan rates in pH7 PBS, under N_2_-saturated conditions (Fig. 3a,b). A pair of well-defined redox peaks were observed at −0.49 V for GOx/MXene/Nafion/GCE. A shift in redox peak towards lower potential is observed for GOx/Au/MXene/Nafion/GCE indicating facilitated electron communication between the active site of GOx and the Mxene electrode with Au decoration (Fig. S2 ESI). Again a linearity of cathodic/anodic peak currents with scan rates (insets of Fig. 3) indicates a surface-confined electrode reaction process.

**Figure 4:**
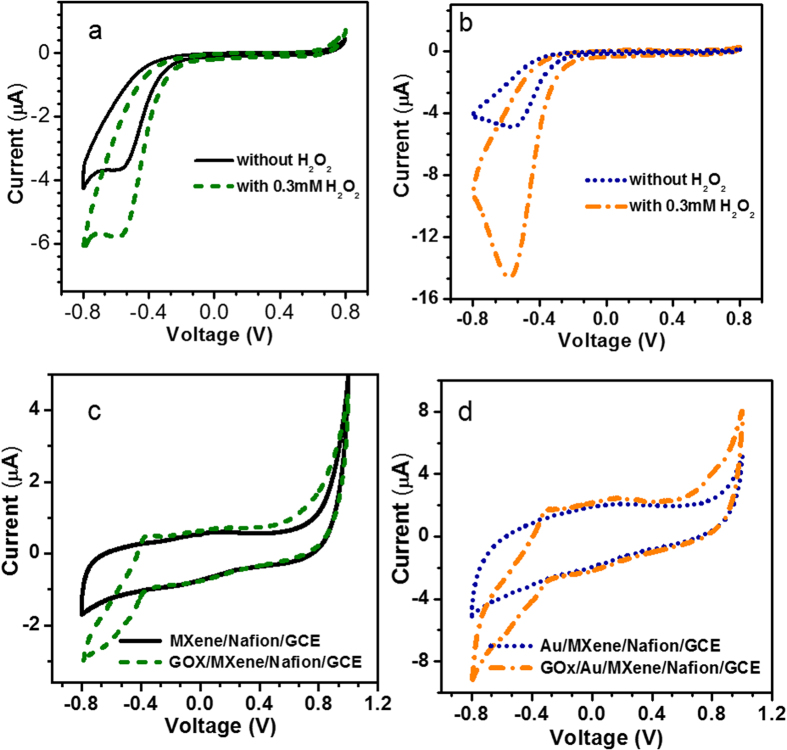
CV responses of the fabricated (**a**) GOx/MXene/Nafion/GCE and (**b**) GOx/Au/MXene/Nafion/GCE bioelectrodes toward the addition of 0.3 mM of H_2_O_2_ to pH 7 phosphate buffer solution at a scan rate of 10 mV/s, CV responses of (**c**) GOx/MXene/Nafion/GCE and (**d**) GOx/Au/MXene/Nafion/GCE bioelectrodes in 5 mM of glucose solution.

The CV curves of the fabricated GOx/MXene/Nafion/GCE and GOx/Au/MXene/Nafion/GCE bioelectrodes to the addition of 0.3 mM of H_2_O_2_ to pH 7 phosphate buffer solution at a scan rate of 10 mV/s are illustrated in Fig. 4a,b respectively. Minor reduction peaks were observed at −0.5 V for both the fabricated electrodes in the absence of H_2_O_2_. In both cases,a significant improvement in the reduction peak current is observed upon the addition of H_2_O_2_. The catalytic activity of Nafion solubilized MXene is identified from Fig. 4a. The reduction current obtained for the GOx/Au/MXene/Nafion/GCE bioelectrode is nearly 3-fold higher than that obtained for GOx/MXene/Nafion/GCE, and this can be attributed to the superior conductivity and catalytic activity of the former due to the incorporation of Au nanoparticles. The remarkable electronic properties induced by the Au nanoparticles in the Au/MXene nanocomposite, results in relatively fast charge transport within the GOx/Au/MXene/Nafion/GCE bioelectrodes, leading to better electrocatalytic performance toward the detection of H_2_O_2_. The direct electrochemistry of GOx immobilized MXene/Nafion/GCE and Au/MXene/Nafion/GCE bioelectrodes were investigated in 5 mM glucose solution. Figure 4c shows the CV curves at the scan rate of 10 mV/s for MXene/Nafion/GCE and GOx/MXene/Nafion/GCE in 5 mM of glucose solution. No significant peaks are observed in the CV loop of the MXene/Nafion/GCE, whereas, the GOx/MXene/Nafion/GCE bioelectrode exhibited a well-defined glucose oxidation peak −0.402 V which is characteristic peak of reversible electron transfer process of redox active center in the GOx during the oxidation of glucose. A similar trend is observed in GOx/Au/MXene/Nafion/GCE electrode in 5 mM of glucose solution andthat is evident from Fig. 4d. Accordingly, the sensing mechanism of glucose can be explained as follows:

GOx enzyme comprises of two identical protein subunits and one flavin adenine dinucleotide (FAD) coenzyme molecule. The FAD coenzyme molecule is present in the active site of the GOxenzyme. Due to its highly reversible electrochemistry, FAD works efficiently as a cofactor. FAD can be reduced in a two-electron, two proton process to form FADH_2_. During the enzymatic reaction between GOx and glucose, glucose is oxidized to glucono-d-lactone and the FAD is reduced to FADH_2_. Subsequently, FADH_2_ undergoes oxidation by dissolved O_2_ producing H_2_O_2_ and becomes FAD^27^. These processes can be represented using the following equations.


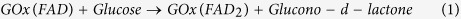



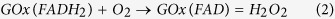


Combining these two equations the oxidation process of glucose in the presence of GOx enzyme can be represented as


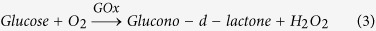


GOx/Au/MXene/Nafion/GCE exhibits better catalytic activity than GOx/ MXene/Nafion/GCE which is evident from the larger value of the peak current.

### Electro catalytic activity of the fabricated biosensors

**Figure 5:**
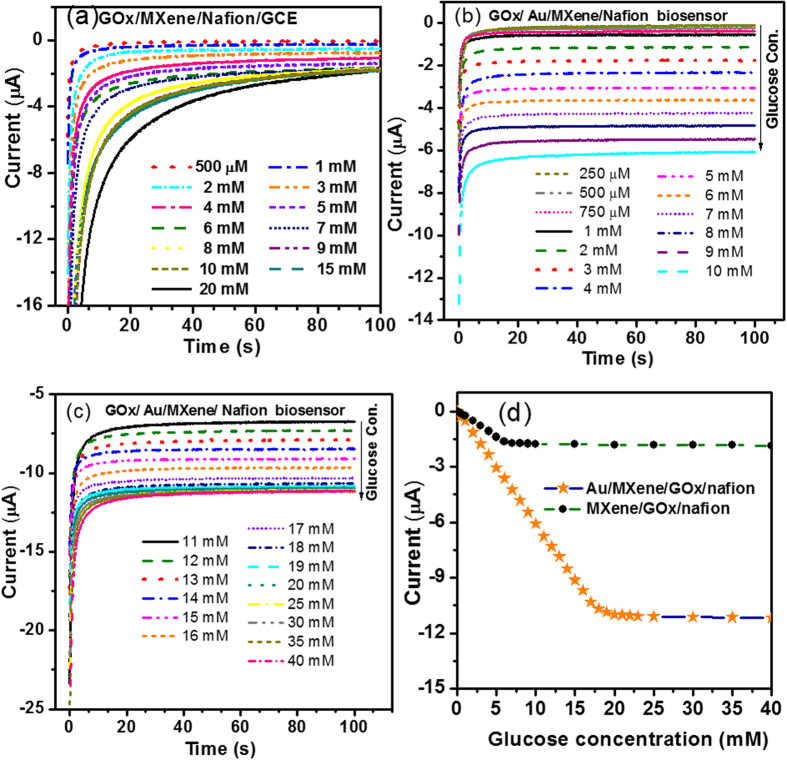
Amperometric *i*-*t* curves for (**a**) GOx/MXene/Nafion/GCE, (**b**,**c**) GOx/Au/MXene/Nafion/GCE at a constant voltage of −0.402 V. (**d**) Steady-state calibration curves for GOx/MXene/Nafion/GCE and GOx/Au/MXene/Nafion/GCE.

Amperometric i-t measurements can be used as an effective tool for investigating the sensitivity of the fabricated biosensor towards varying conc. of glucose in the supporting electrolyte. Amperometric*i*-*t*curves for the MXene/Nafion/GCE at a constant voltage of −0.402 V is shown in Fig. 5a. The amperometric response of the fabricated bioelectrode toward the detection of glucose has been carefully recorded by increasing the concentration of glucose in the 0.1 M phosphate buffer (pH 7) solution systematically. Figure 5b,c show typical amperometric i-t curves recorded for the fabricated GOx/Au/MXene/Nafion/GCE bioelectrode. For both GOx/MXene/Nafion/GCE and GOx/Au/MXene/Nafion/GCE, at a particular conc. of glucose, a stable current is achieved after a period. For GOx/MXene/Nafion/GCE, at lower concentration of glucose, the current stability is achieved within 20 s. Whereas, for GOx/Au/MXene/Nafion/GCE bioelectrode, at lower glucose concentration, a stable value is reached within 10 s. A general trend of increasing current with increasing glucose concentration is observed from all the three graphs (Fig. 5a–c). This clearly demonstrates the sensitivity of the fabricated biosensors to varying glucose concentration. Figure 5d shows the steady-state calibration curves drawn using the steady-state current values corresponding to different glucose concentrations for GOx/MXene/Nafion/GCE and GOx/Au/MXene/Nafion/GCE. The steady-state calibration curve for GOx/MXene/Nafion/GCE biosensor exhibits a linear range from 0.5–6 mM conc. of glucose with a detection limit of 100 μM and that of GOx/Au/MXene/Nafion/GCE exhibits a linear range from 0.1–18 mM conc. of glucose with a detection limit of 5.9 μM (S/N = 3). GOx/Au/MXene/Nafion/GCE biosensor exhibited a sensitivity of 4.2 μAmM^−1^cm^−2^.

Storage stability of the GOx/Au/MXene/Nafion/GCE biosensor was investigated by every day monitoring its response current to 3 mM glucose conc. The biosensor electrode retained 93% of its initial response current even after two months storage period indicating its good storage stability. The electrode also exhibited excellent repeatability (twelve repeated measurements) and reproducibility (twelve individual measurements) with an average relative standard deviation of 1.26% and 2.74%, respectively.

The selectivity of the fabricated biosensor electrode is assessed by investigating the influence of the common interfering electroactive substances such as dopamine (DA), uric acid (UA) and ascorbic (AA) and the results are presented in Fig. S3. The fabricated GOx/Au/MXene/Nafion/GCE biosensor electrode, shows better selectivity towards the detection of glucose as compared to DA, UA or AA at −0.402 V, which can be attributed to the presence of negatively charged Nafion solution coating on the surface of the electrode.

## Discussions

Since MXene nanosheets have excellent in-plane conductivity, when used as an enzyme immobilization matrix, both MXene and Au/MXene are capable of improving electron transfer kinetics between the active redox centers of the enzyme. Nafion assists the dispersion of Au/MXene composite, whereby the Au/MXene remain well-dispersed in prolonged standing and helps in forming uniform and stable film on the GCE. The superior sensing performance of GOx/Au/MXene/Nafion/GCE is attributed to the improvement in the relative activity of GOx in the presence of Au nanoparticles. It has been widely reported that the biosensing properties of graphene-based sensors can be dramatically improved by anchoring Au nanoparticles on the surface of graphene nanosheets^53^,^54^,^55^,^56^,^57^. In a similar way, dispersion of Au nanoparticles on the surface of MXene nanosheets is expected to improve the electrical conductivity and makes the resultant Au/MXene composite as a better electrochemical transducer/enzyme immobilization matrix. Furthermore, Nafion being a negatively charged polyelectrolyte matrix, reduces the permeability of negatively charged substrates. Hence the Nafion coating on the GOx/Au/MXene/Nafion/GCE surface eliminates the influence of interfering signals and improves the selectivity of the sensor.

Table 1 shows a comparison of some of the relevant analytical parameters such as detection limit, linear range and sensitivity of the fabricated biosensors in the present study with literature results. It is evident from this table that the GOx/Au/MXene/Nafion/GCE bioelectrode exhibits significantly improved sensing performance than most of the previously reported glucose biosensors. The performance of the biosensor is comparable to some of the best published performances.

The present study successfully demonstrated for the first time, the use of Nafion solubilized Au/MXene nanocomposite as a novel matrix for GOx immobilization. GOx/Au/MXene/Nafion/GCE biosensor is fabricated by a mere dropcasting method. Au nanoparticles anchored on the surface of MXene nanosheets significantly improved the electron transfer process between GOx and GCE. The fabricated biosensor electrode exhibited excellent electrocatalytic activity toward the detection of glucose with a wide linear range from 0.1 mM to 18 mM, good sensitivity of 4.2 μA mM^−1^ cm^−2^, a lower detection limit of 5.9 μM, excellent stability, repeatability and reproducibility. The results indicate that the GOx/Au/MXene/Nafion/GCE biosensor electrode is suitable for measurement of the glucose conc. (in a range 0.1–18 mM) in biological samples for the detection of Diabetes Mellitus.

## Methods

### Materials


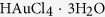
 D-(+)-Glucose and Hydrogen Peroxide solution were purchased from Sigma-Aldrich. The glucose stock solution was allowed to mutarotate for 24 h at room temperature prior to use and subsequently store at 4 °C. Nafion solution (D1021 Water based 1100 EW at 10% weight) and GOx (EC 1.1.3.4, Aspergillus niger, >100 U/mg) were purchased from Ion Power Inc. and Alfa Aesar respectively. Ti_3_C_2_T_x_ MXenes were synthesized by chemical vapor deposition technique as described below in section 2.2. The supporting electrolyte was 0.1 M phosphate buffer at pH 7 unless otherwise stated. The common chemicals used for the preparation of buffers, etc., were of analytical reagent grade. All of the solutions were prepared with deionized distilled (DI) water.

### Synthesis of Ti_3_C_2_T_x_ MXene

Two-dimensional titanium carbide nanosheets were synthesized by exfoliation of commercially procured Ti_3_AlC_2_ powders. The as-received Ti_3_AlC_2_ powders were immersed in 40% HF for 2.5 h at room temperature. The resulting suspension was collected and then washed with DI water for several times and then filtered to get 2D Titanium Carbide nanosheets (MXenes).

### Preparation of Au/MXene Composite

In order to decorate the Ti_3_C_2_T_x_ MXene nanosheets with nanocrystalline Au clusters, a chemical reduction method was used. 0.075 M HAuCl_4_·3H_2_O solution is reduced by adding a reducing solution, which is a mixture of 0.1 M NaBH_4_ and 1 M NaOH, assisted by stirring. Nearly 0.05 g of Ti_3_C_2_T_X_ MXene was added to the above solution. The resultant mixture is subjected to ultrasonication for 30 minutes. Once, the reaction is over; the solution is washed three times with deionized water and filtered using cellulose membrane filters having a pore size of 0.1 μm. The material left out is collected and dried in a vacuum oven at 80 °C for 2 h. The sample was then characterized using XRD, SEM, and energy dispersive analysis of X-rays (EDAX).

### Fabrication of GOx/Au/MXene/Nafion/GCE

In order to get a clean mirror surface, the bare GCE was polished with 0.05 μm alumina slurry, sonicated in deionized water, and dried with a high-purity nitrogen stream. The Ti_3_C_2_T_X_ MXene (or Au/MXene nanocomposite) was sonicated in 0.5% Nafion solution to get a concentration of ∼1 mg/ml. 4 μL of the suspension was then film cast onto the surface of the GCE and allowed to dry slowly. A 4 *μ*L portion of 32 U GOx solution was film cast onto the surface of the Au/MXene/Nafion/GCE and allowed to dry slowly at 4 °C. The obtained GOx/Au/MXene/Nafion/GCE electrode was washed carefully with doubly distilled water and dried at less than 4 °C. These GOx/Au/MXene/Nafion/GCE bioelectrodes were coated with an extra 2 *μ*L layer of 0.5% Nafion. The electrodes were rinsed with pH 7 buffer and stored in the buffer at 4 °C prior to use.

### Instruments

The electrochemical measurements were performed using a CH Instruments CHI 660D Electrochemical Analyzer/Workstation. A Pt wire counter electrode, Ag/AgCl (3 M KCl) reference electrode, and glassy carbon electrode (GCE, diameter 3 mm) were inserted into a modified 10 mL glass cell (Model CHI-222) for the measurement. All potentials are referred to the Ag/AgCl reference electrode. A magnetic stirrer provided the convective transport at 300 rpm during the amperometric measurements, and the background current was allowed to decay to a steady-state value before spiking the equilibrated -D-glucose. The nanocomposite materials were characterized by a powder X-ray diffraction system (XRD, Bruker, D8 ADVANCE) equipped with Cu Kα radiation (λ = 0.15406 nm). The surface morphology and microstructure of the samples were investigated by a scanning electron microscopy (SEM, FEI Quanta 600D and NOVA NANO SEM) and transition electron microscopy (TEM, FEI Titan) and the elemental presence was identified using energy dispersive X-ray analysis (EDAX).

## Additional Information

**How to cite this article**: Rakhi, R. B. *et al*. Novel amperometric glucose biosensor based on MXene nanocomposite. *Sci. Rep*. **6**, 36422; doi: 10.1038/srep36422 (2016).

**Publisher’s note**: Springer Nature remains neutral with regard to jurisdictional claims in published maps and institutional affiliations.

## Supplementary Material

Supplementary Information

## Figures and Tables

**Table 1:** Comparison of analytical performance of the fabricated GOx/Au/MXene/Nafion/GCE biosensor with other glucose biosensor.

Electrode	Linear range (mM)	Detection Limit (μM)	Sensitivity (μA mM^−1^ cm ^−2^)	Reference
GOx/Au/MWCNT/Pt	0.1–10	6.7	2.57	58
GOx/MWCNTs/CS/GCE	0–7.8	10	0.52	59
PDDA–GOx/Au/MWCNTs/GCE	0.5–5	—	3.96	60
GOx/graphene/PANI/Au/GCE	0.004–1.12	0.6	—	61
GOx/PPyAA/Au/GCE	1–18	50	0.42	22
G-MWCNT/GCE	6.30–20.09	—	2.47	62
GOx/GR-MWNTs/AuNPs	0.01–2 2–5.2	4.1 950	0.695 0.2380	21
GOx/Au/MWCNTs/PVA/GCE	0.5–8	200	16.6	63
*GOx/Au/MXene/Nafion/GCE*	*0.1*–*18*	*5.9*	*4.2*	*This work*

## References

[b1] Naguib M. . Two-Dimensional Transition Metal Carbides. ACS nano 6, 1322–1331 (2012). 22279971 10.1021/nn204153h

[b2] Naguib M. . Two-Dimensional Nanocrystals Produced by Exfoliation of Ti_3_AlC_2_. Adv. Mater. 23, 4248–4253 (2011). 21861270 10.1002/adma.201102306

[b3] Naguib M., Mochalin V. N., Barsoum M. W. & Gogotsi Y. 25th Anniversary Article: MXenes: A New Family of Two-Dimensional Materials. Adv. Mater. 26, 992–1005 (2014). 24357390 10.1002/adma.201304138

[b4] Hwang J. Y. . Direct preparation and processing of graphene/RuO_2_ nanocomposite electrodes for high-performance capacitive energy storage. Nano Energy 18, 57–70 (2015).

[b5] Lukatskaya M. R. . Cation Intercalation and High Volumetric Capacitance of Two-Dimensional Titanium Carbide. Science 341, 1502–1505 (2013). 24072919 10.1126/science.1241488

[b6] Naguib M. . MXene: a promising transition metal carbide anode for lithium-ion batteries. Electrochem. Commun. 16, 61–64 (2012).

[b7] Dall’Agnese Y. . High capacitance of surface-modified 2D titanium carbide in acidic electrolyte. Electrochem. Commun. 48, 118–122 (2014).

[b8] Tang Q., Zhou Z. & Shen P. Are MXenes Promising Anode Materials for Li Ion Batteries? Computational Studies on Electronic Properties and Li Storage Capability of Ti_3_C_2_ and Ti_3_C_2_X_2_ (X = F, OH) Monolayer. J. Am. Chem. Soc. 134, 16909–16916 (2012). 22989058 10.1021/ja308463r

[b9] Wang F. . An Organ-Like Titanium Carbide Material (MXene) with Multilayer Structure Encapsulating Hemoglobin for a Mediator-Free Biosensor. J. Electrochem. Soc. 162, B16–B21 (2015).

[b10] Yu Z. . Facile synthesis of NiCo_2_O_4_@Polyaniline core-shell nanocomposite for sensitive determination of glucose. Biosensors & bioelectronics 75, 161–165 (2016). 26318785 10.1016/j.bios.2015.08.024

[b11] Sedghi R. & Pezeshkian Z. Fabrication of non-enzymatic glucose sensor based on nanocomposite of MWCNTs-COOH-Poly(2-aminothiophenol)-Au NPs. Sens. Actuators 219, 119–124 (2015).

[b12] Mani V. . Electrodeposition of copper nanoparticles using pectin scaffold at graphene nanosheets for electrochemical sensing of glucose and hydrogen peroxide. Electrochim. Acta 176, 804–810 (2015).

[b13] Khan M. & Park S.-Y. Glucose biosensor based on GOx/HRP bienzyme at liquid-crystal/aqueous interface. J. Colloid Interface Sci. 457, 281–288 (2015). 26196711 10.1016/j.jcis.2015.07.010

[b14] Chou J.-C. . Dynamic and Wireless Sensing Measurements of Potentiometric Glucose Biosensor Based on Graphene and Magnetic Beads. IEEE Sens. J. 15, 5718–5725 (2015).

[b15] Balouch Q. . Cobalt Oxide Nanoflowers for Electrochemical Determination of Glucose. J. Electron. Mater. 44, 3724–3732 (2015).

[b16] Li C. L., Wang H. J. & Yamauchi Y. Electrochemical Deposition of Mesoporous Pt-Au Alloy Films in Aqueous Surfactant Solutions: Towards a Highly Sensitive Amperometric Glucose Sensor. Chemistry-a European Journal 19, 2242–2246 (2013). 10.1002/chem.201203378 23307536

[b17] Malgras V. . Nanoarchitectures for Mesoporous Metals. Adv. Mater. 28, 993–1010 (2016). 26515804 10.1002/adma.201502593

[b18] Martinkova P. & Pohanka M. Biosensors for blood glucose and diabetes diagnosis: evolution, construction, and current status. Anal. Lett. 48, 2509–2532 (2015).

[b19] Korotkaya E. V. Biosensors: design, classification, and applications in the food industry. Foods and Raw Materials 2, 161–171 (2014).

[b20] Glen E. & Fryxell G. C. Environmental Applications of Nanomaterials: Synthesis, Sorbents and Sensors. 2 edn, (Imperial College Press, 2012).

[b21] Devasenathipathy R. . Glucose biosensor based on glucose oxidase immobilized at gold nanoparticles decorated graphene-carbon nanotubes. Enzyme Microb. Technol. 78, 40–45 (2015). 26215343 10.1016/j.enzmictec.2015.06.006

[b22] Senel M. & Nergiz C. Novel amperometric glucose biosensor based on covalent immobilization of glucose oxidase on poly(pyrrole propylic acid)/Au nanocomposite. Curr. Appl Phys. 12, 1118–1124 (2012).

[b23] Samphao A. . Flow-injection amperometric determination of glucose using a biosensor based on immobilization of glucose oxidase onto Au seeds decorated on core Fe_3_O_4_ nanoparticles. Talanta 142, 35–42 (2015). 26003689 10.1016/j.talanta.2015.01.046

[b24] Ghasemi E., Shams E. & Nejad N. F. Covalent modification of ordered mesoporous carbon with glucose oxidase for fabrication of glucose biosensor. J. Electroanal. Chem. 752, 60–67 (2015).

[b25] Chengcheng L. . A glucose oxidase-coupled DNAzyme sensor for glucose detection in tears and saliva. Biosens. Bioelectron. 70, 455–461 (2015). 25863343 10.1016/j.bios.2015.03.070

[b26] Heller A. & Feldman B. Electrochemical glucose sensors and their applications in diabetes management. Chem. Rev. 108, 2482–2505 (2008). 18465900 10.1021/cr068069y

[b27] Harper A. & Anderson M. R. Electrochemical Glucose Sensors-Developments Using Electrostatic Assembly and Carbon Nanotubes for Biosensor Construction. Sensors 10, 8248–8274 (2010). 22163652 10.3390/s100908248 PMC3231221

[b28] Palod P. A. & Singh V. Improvement in glucose biosensing response of electrochemically grown polypyrrole nanotubes by incorporating crosslinked glucose oxidase. Materials Science & Engineering C-Materials for Biological Applications 55, 420–430 (2015). 10.1016/j.msec.2015.05.038 26117773

[b29] Anusha J. R. . Amperometric glucose biosensor based on glucose oxidase immobilized over chitosan nanoparticles from gladius of Uroteuthis duvauceli. Sens. Actuators, B-Chemical 215, 536–543 (2015).

[b30] Tembe S. & D’Souza S. F. Immobilisation strategies for construction of tyrosinase-based biosensors. Mater. Technol. 30, B190–B195 (2015).

[b31] Hossain M. F., Heo M., Shin J. H. & Park J. Y. An Electrochemical Enzymatic Biosensor Based on Au/FGs/sol-gel-GOx Composite/Nafion. Int. J. Electrochem. Sci. 10, 6803–6819 (2015).

[b32] Anusha J. R. . Effective immobilization of glucose oxidase on chitosan submicron particles from gladius of Todarodes pacificus for glucose sensing. Bioelectrochemistry 104, 44–50 (2015). 25727854 10.1016/j.bioelechem.2015.02.004

[b33] Nenkova R., Wu J., Zhang Y. & Godjevargova T. Evaluation of Immobilization Techniques for the Fabrication of Nanomaterial-Based Amperometric Glucose Biosensors. Anal. Lett. 48, 1297–1310 (2015).

[b34] Yang Z. . An enzymatic glucose biosensor based on a glassy carbon electrode modified with cylinder-shaped titanium dioxide nanorods. Microchimica Acta 182, 1841–1848 (2015).

[b35] Baghayeri M. Glucose sensing by a glassy carbon electrode modified with glucose oxidase and a magnetic polymeric nanocomposite. Rsc Advances 5, 18267–18274 (2015).

[b36] Xin D. . Facile synthesis of beta-lactoglobulin-functionalized multi-wall carbon nanotubes and gold nanoparticles on glassy carbon electrode for electrochemical sensing. Biosens. Bioelectron. 62, 73–78, doi: 10.1016/j.bios.2014.06.030 (2014). 24984286

[b37] Baby T. T., Aravind S. S. J., Arockiadoss T., Rakhi R. B. & Ramaprabhu S. Metal decorated graphene nanosheets as immobilization matrix for amperometric glucose biosensor. Sens. Actuators 145, 71–77 (2010).

[b38] Nayak P., Santhosh P. N. & Ramaprabhu S. Synthesis of Au-MWCNT-Graphene hybrid composite for the rapid detection of H_2_O_2_ and glucose. Rsc Advances 4, 41670–41677 (2014).

[b39] Zhong G.-X. . A nonenzymatic amperometric glucose sensor based on three dimensional nanostructure gold electrode. Sens. Actuators 212, 72–77 (2015).

[b40] Chowdhury A. D., Gangopadhyay R. & De A. Highly sensitive electrochemical biosensor for glucose, DNA and protein using gold-polyaniline nanocomposites as a common matrix. Sens. Actuators 190, 348–356 (2014).

[b41] Qin X. . Graphene/polyaniline/gold nanoparticles nanocomposite for the direct electron transfer of glucose oxidase and glucose biosensing. Sens. Actuators, B (Chemical) 190, 562–569 (2014).

[b42] Qingping W., Fanfei M. & Jinbo Z. Preparation of gold nanowires and its application in glucose biosensing. Mater. Lett. 91, 9–11 (2013).

[b43] Fakhrabadi N. H., Rohani A. A. & Farahbakhsh A. In Functional Materials and Nanotechnology Vol. 496 Advanced Materials Research (eds Xu B. & Li H. Y.) 17–20 (2012).

[b44] Li C. . Electrochemical synthesis of mesoporous gold films toward mesospace-stimulated optical properties. Nat. Commun. 6, doi: 10.1038/ncomms7608 (2015). PMC4382992 25799072

[b45] Li C. L. . Superior electrocatalytic activity of mesoporous Au film templated from diblock copolymer micelles. Nano Research 9, 1752–1762 (2016).

[b46] Dong X. C. . 3D Graphene-Cobalt Oxide Electrode for High-Performance Supercapacitor and Enzymeless Glucose Detection. Acs Nano 6, 3206–3213 (2012). 22435881 10.1021/nn300097q

[b47] Zhang M. . Highly sensitive glucose sensors based on enzyme-modified whole-graphene solution-gated transistors. Sci. Rep. 5, doi: 10.1038/srep08311 (2015). PMC4319171 25655666

[b48] Hvolbaek B. . Catalytic activity of Au nanoparticles. Nano Today 2, 14–18 (2007).

[b49] Shaowen Cao F. F. T., Yu Tang, Yuting Li & Jiaguo Yu. Size and shape dependent catalytic performances of oxidation and reduction reactions on nanocatalysts. Chem. Soc. Rev., doi: 10.1039/C6CS00094K (2016). 27276189

[b50] Ishida T. . Influence of the Support and the Size of Gold Clusters on Catalytic Activity for Glucose Oxidation. Angew. Chem. Int. Ed. 47, 9265–9268 (2008). 10.1002/anie.200802845 18850617

[b51] Rakhi R. B., Sethupathi K. & Ramaprabhu S. A Glucose Biosensor Based on Deposition of Glucose Oxidase onto Crystalline Gold Nanoparticle Modified Carbon Nanotube Electrode. J. Phys. Chem. 113, 3190–3194 (2009). 10.1021/jp810235v 19260716

[b52] Zargoosh K. . Highly sensitive glucose biosensor based on the effective immobilization of glucose oxidase/carbon-nanotube and gold nanoparticle in nafion film and peroxyoxalate chemiluminescence reaction of a new fluorophore. Talanta 93, 37–43 (2012). 22483873 10.1016/j.talanta.2011.11.029

[b53] Valappil M. O., Alwarappan S. & Narayanan T. N. Atomic Layers in Electrochemical Biosensing Applications - Graphene and Beyond. Curr. Org. Chem. 19, 1163–1175 (2015).

[b54] Aswathi R., Ali M. M., Shukla A. & Sandhya K. Y. A green method to gold-graphene nanocomposite from cyclodextrin functionalized graphene for efficient non-enzymatic electrochemical sensing applications. Rsc Advances 5, 32027–32033 (2015).

[b55] Wang L. . Highly Sensitive Electrochemical Biosensor for Evaluation of Oxidative Stress Based on the Nanointerface of Graphene Nanocomposites Blended with Gold, Fe_3_O_4_, and Platinum Nanoparticles. ACS Appl. Mater. Interfaces 7, 18441–18449 (2015). 26238430 10.1021/acsami.5b04553

[b56] Chu Z. . *In-situ* fabrication of well-distributed gold nanocubes on thiol graphene as a third-generation biosensor for ultrasensitive glucose detection. Electrochim. Acta 176, 162–171 (2015).

[b57] Li S. . A novel hydrogen peroxide sensor based on Ag nanoparticles decorated polyaniline/graphene composites. J. Appl. Polym. Sci. 132, doi: 10.1002/app.42409 (2015).

[b58] Wu B.-Y. . Amperometric glucose biosensor based on multilayer films via layer-by-layer self-assembly of multi-wall carbon nanotubes, gold nanoparticles and glucose oxidase on the Pt electrode. Biosensors & Bioelectronics 22, 2854–2860 (2007). 17212983 10.1016/j.bios.2006.11.028

[b59] Deng S., Jian G., Lei J., Hu Z. & Ju H. A glucose biosensor based on direct electrochemistry of glucose oxidase immobilized on nitrogen-doped carbon nanotubes. Biosensors & Bioelectronics 25, 373–377 (2009). 19683424 10.1016/j.bios.2009.07.016

[b60] Yao Y.-L. & Shiu K.-K. Direct electrochemistry of glucose oxidase at carbon nanotube-gold colloid modified electrode with poly(diallyldimethylammonium chloride) coating. Electroanalysis 20, 1542–1548 (2008).

[b61] Xu Q. . Graphene/polyaniline/gold nanoparticles nanocomposite for the direct electron transfer of glucose oxidase and glucose biosensing. Sens. Actuators, B-Chemical 190, 562–569 (2014).

[b62] Periasamy A. P., Chang Y.-J. & Chen S.-M. Amperometric glucose sensor based on glucose oxidase immobilized on gelatin-multiwalled carbon nanotube modified glassy carbon electrode. Bioelectrochem. 80, 114–120 (2011). 10.1016/j.bioelechem.2010.06.009 20638345

[b63] Zhang H., Meng Z., Wang Q. & Zheng J. A novel glucose biosensor based on direct electrochemistry of glucose oxidase incorporated in biomediated gold nanoparticles-carbon nanotubes composite film. Sens. Actuators, B-Chemical 158, 23–27 (2011).

