# Erratum: Novel amperometric glucose biosensor based on MXene nanocomposite

**DOI:** 10.1038/srep38465

**Published:** 2016-12-09

**Authors:** R. B. Rakhi, Pranati Nayak, Chuan Xia, Husam N. Alshareef

Scientific Reports 6: Article number: 3642210.1038/srep36422; published online: 11 10 2016; updated: 12 09 2016

The original version of this Article contained a typographic error in the spelling of Pranati Nayak, which was incorrectly given as Pranati Nayuk.

This error has now been fixed in the HTML and PDF versions of this Article.

