# Supplementary material for: Novel amperometric glucose biosensor based on MXene nanocomposite
Source: Sci Rep. 2016 Nov 10;6:36422. doi: 10.1038/srep36422 (PMC5103228; doi:10.1038/srep36422)
Supplement: Supplementary Information [file srep36422-s1.pdf]

Supporting Information

Novel amperometric glucose biosensor based on MXene nanocomposite

R.B. Rakhi[1, 2, †], P. Nayak[1,†], C. Xia[1], and Husam N. Alshareef[1,*]

[1]Materials Science and Engineering, King Abdullah University of Science and Technology,(KAUST), Thuwal 23955-6900, Saudi Arabia,

[2]Chemical Sciences and Technology Division, CSIR-National Institute of Interdisciplinary Science and Technology (CSIR-NIIST), Thiruvananthapuram, 695019, India

---

†Authors contributed equally to this work.

*Corresponding author: husam.alshareef@kaust.edu.sa

Phone: Office: +966-(0)2-808-4477 | Cell: +966-(0)5-44700037

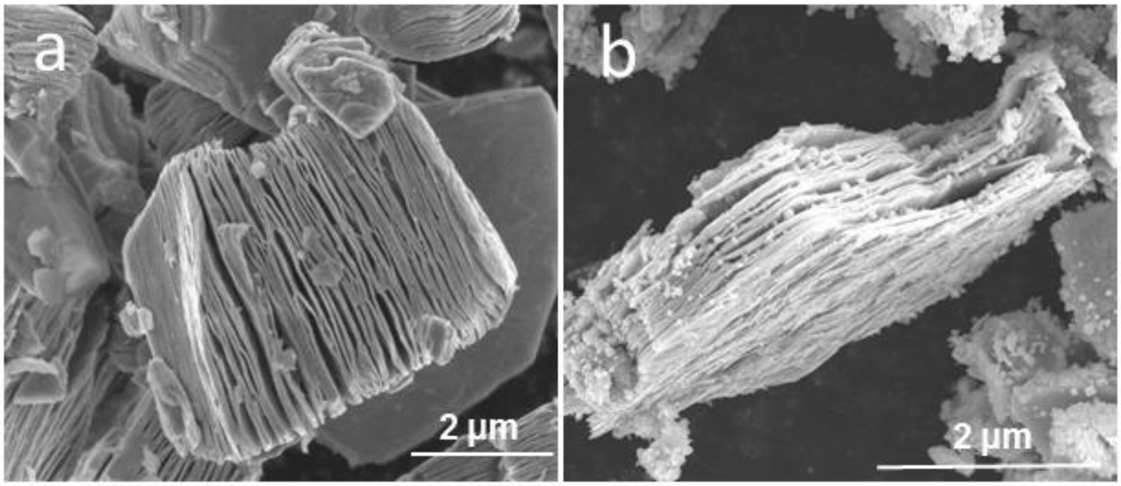

Figure S1: SEM images of (a) MXene and (b) Au/MXene composite

FESEM images provide a better understanding of the morphology of Au/MXene nanocomposite. SEM image of the $Ti_3C_2T_x$ MXene (Fig. S1a) shows a structure composed of stacked 2D MXene nanosheets, where the individual layers are separated from each other, resulting in a morphology similar to that of exfoliated graphite. From the SEM images of the Au/MXene nanocomposites shown in Fig. S2b, it is evident that the Au nanoparticles are evenly distributed on the surface and in between the layers of MXene nanosheets, which can result in an improvement in the in sheet as well as the inter-layer conductivity. The original structure of the MXene stack is retained even after the introduction of Au nanoparticles.

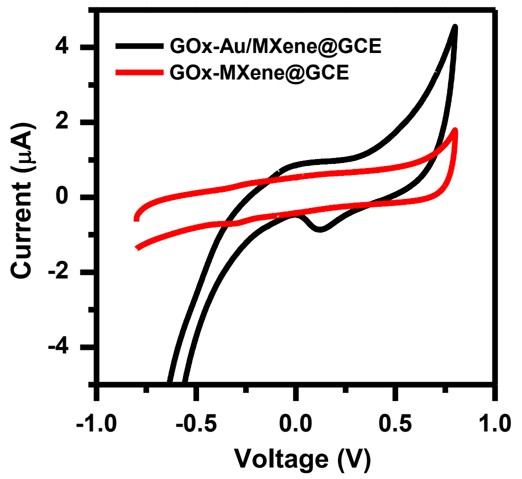

Figure S2: Cyclic voltammograms of GOx/Mxene/Nafion/GCE and Gox-Au/Mxene/Nafion/GCE electrodes in N_2-saturated pH 7 PBS.

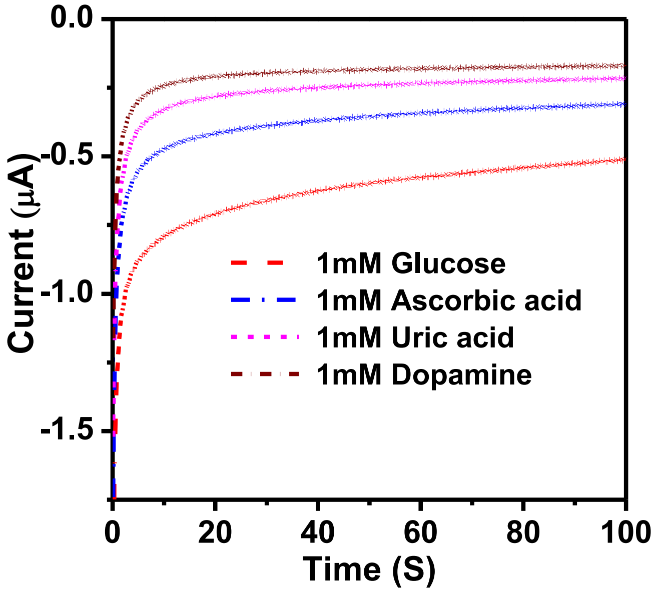

Figure s3: Interference studies of GOx/Au/Mxene/Nafion/GCE
